# Cutaneous *Scedosporium apiospermum* infection in a patient with metastatic renal cell carcinoma

**DOI:** 10.1016/j.jdcr.2024.07.013

**Published:** 2024-08-03

**Authors:** Goranit Sakunchotpanit, Thomas Z. Rohan, Jakob Moran, Eleanor Russell-Goldman, Michelle Walters, Vinod E. Nambudiri

**Affiliations:** a Department of Dermatology, Brigham and Women’s Hospital, Boston, Massachusetts; b Tufts University School of Medicine, Boston, Massachusetts; c Sidney Kimmel Medical College, Thomas Jefferson University, Philadelphia, Pennsylvania; d Department of Pathology, Brigham and Women’s Hospital, Boston, Massachusetts; e Harvard Medical School, Boston, Massachusetts

**Keywords:** cutaneous *Scedosporium*, fungal infection, immunocompromised, metastatic renal cell carcinoma, voriconazole

## Introduction

*Scedosporium apiospermum* (also known as *Pseudallescheria boydii*) is a rare, opportunistic fungal pathogen found in soil, contaminated water, and bird droppings.^1^ The primary route of entry is either through spore inhalation or traumatic inoculation.^1^ Although infection in the immunocompetent is typically limited to the superficial skin, immunosuppression has been documented to promote systemic manifestations via hematogenous spread.^1^ Notably, patients on immunosuppressants have experienced sinopulmonary and ocular symptoms, with higher fungal burdens resulting in central nervous system involvement.^1^ Combined with reported resistance to amphotericin B and antifungal azoles in some isolates, rapid identification of this pathogen is imperative to the management of susceptible patients.^1^^,^^2^ Without adequate awareness, practicing dermatologists run the risk of confusing *S apiospermum* for other, more common filamentous molds.^1^^,^^3^

## Case report

A 68-year-old man with type 2 diabetes, stage III chronic kidney disease, and pT3N1 fumarate hydratase-deficient renal cell carcinoma presented for dermatologic evaluation of a left forearm eruption. He had known widely metastatic renal cell carcinoma lesions in the left lung, abdominal wall, spine, and lymph nodes, and had been treated with nivolumab-ipilimumab combination immunotherapy (initially 4 cycles of 3 mg/kg nivolumab and 1 mg/kg ipilimumab, followed by maintenance treatment with 480 mg nivolumab every 4 weeks thereafter). The patient had also been prescribed dexamethasone (2 mg twice daily) for malignancy-related pain.

**Fig 1:**
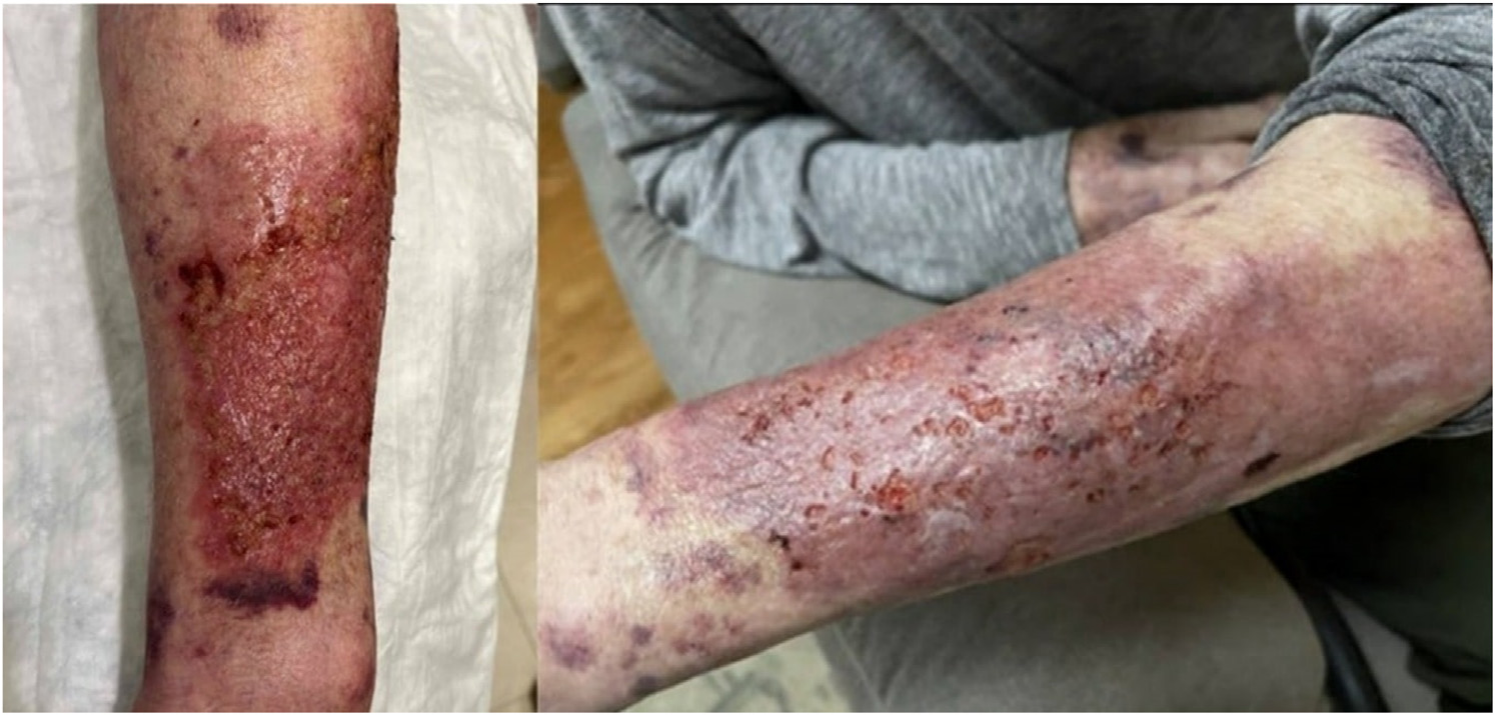
Erythematous, eroded plaque with cribriform scarring and minimal purulent drainage on the left forearm.

The forearm lesion had developed from a painless skin tear 2 months prior, and had progressed to a painful, erythematous plaque over the prior month. Empiric treatment with cephalexin (500 mg twice daily for 7 days) for suspected cellulitis yielded no improvement. Application of topical clobetasol (0.05%) resulted in decreased pain and swelling; however, the lesion persisted and developed drainage, prompting referral for dermatologic evaluation. The patient was afebrile and well-appearing, with an erythematous, eroded plaque on the left forearm with cribriform scarring and minimal purulent drainage (Fig 1). Violaceous ecchymoses were also present throughout the upper extremities. The patient denied a history of similar skin lesions or pathergy at other sites.

**Fig 2:**
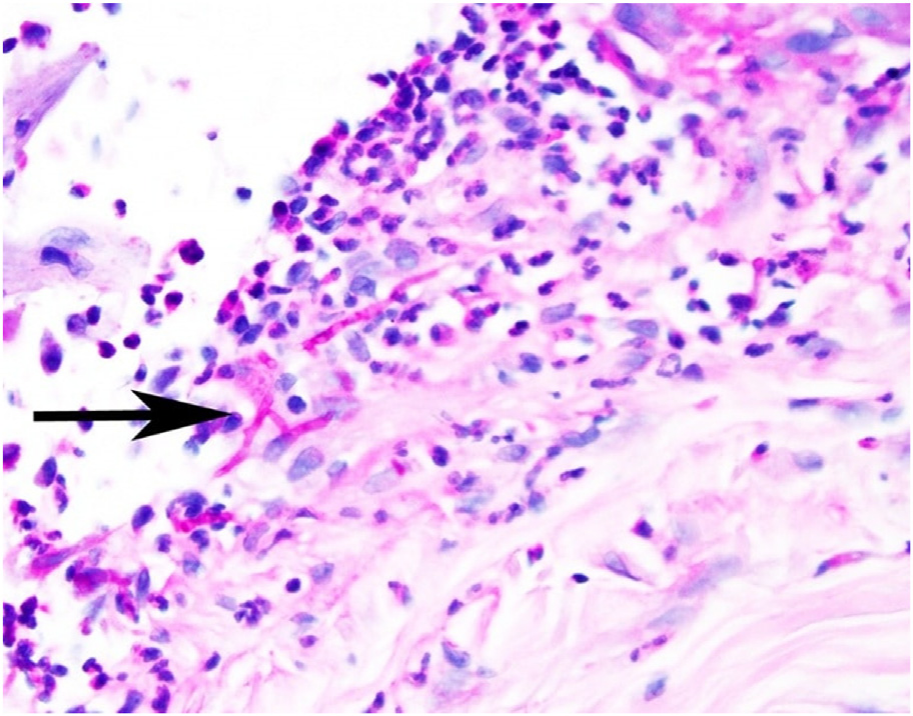
Fungal hyphae (*black arrow*) present on the surface and in the papillary dermal tissue in a background of acute and chronic inflammation (periodic acid–Schiff with diastase; cropped image at ×200 magnification).

Given its appearance, development after trauma, and the patient’s history of immunomodulation, the differential diagnosis included cutaneous infection as well as inflammatory causes (eg, pyoderma gangrenosum). Punch biopsies were performed for histopathology and tissue culture. Histologic analysis demonstrated superficial dermal acute and chronic inflammation, and numerous fungal hyphae were noted (Fig 2). Fungal tissue cultures grew *S apiospermum*; no resistance to common antifungals was reported. On additional history-taking, the patient denied any gardening-related activities, but reported that his home water supply was from a nonmunicipal well source. Blood testing was notable for elevated for 1,3-β-d-glucan levels (185 pg/mL). A chest computed tomography scan confirmed no pulmonary involvement. Oral voriconazole was initiated (400 mg twice daily on the first day, 200 mg twice daily afterward) and his dexamethasone was also tapered (from 4 mg to 2 mg daily). The lesion largely resolved over the next 3 weeks, with no reported adverse events from the medication. However, the patient passed away because of malignancy progression 1 month after *S apiospermum* diagnosis.

## Discussion

With increasing use of corticosteroids, immunosuppressants, antineoplastics, and broad-spectrum antibiotics, the number of cutaneous *S apiospermum* cases have increased in recent years.^1^^,^^3^ Although cases of cutaneous infection have been documented in immunocompetent patients, the vast majority involved the immunocompromised—many of whom were taking immunosuppressive drugs or had chronic illness.^3^^,^^4^ Furthermore, nearly all documented cutaneous *S apiospermum* patients had lesions localized to the extremities, implicating minor skin trauma as the main trigger for infection.^3^ Indeed, in addition to type 2 diabetes, chronic kidney disease, and metastatic cancer, our patient had a history of nivolumab-ipilimumab combination immunotherapy alongside long-term dexamethasone use. A small skin tear also served as the nidus for infection in his left forearm. Additionally, despite the absence of official treatment guidelines, most patients (including ours) were responsive to imidazoles.^3^ Miconazole and voriconazole are among the most efficacious treatments reported in the literature, whereas data for itraconazole are mixed.^3^ Surgical debridement or drainage in addition to antifungal therapy has also seen success in select cases.^5^

Although *S apiospermum* is a rare opportunistic fungus, infection can result in significant clinical morbidity. Being vulnerable to other infectious diseases, several immunocompromised patients with localized skin lesions have died from complications, such as bacterial pneumonia and systemic fungal infection.^3^ To help mitigate disease burden, several actions can be taken empirically to promote prompt recognition and treatment initiation. For instance, upon being consulted on an immunocompromised patient, dermatologists should be encouraged to take a detailed history, focusing on a patient’s daily activities of living. Several activities (eg, gardening and walking barefoot) have been linked to initial *S apiospermum* infection; therefore, a detailed history can prompt timely physical examination.^3^ Moreover, it should be noted that *S apiospermum* can present differently than the lesion recognized in our patient. Cutaneous infection has been documented to mimic other filamentous fungi (eg, *Aspergillus* spp), presenting with ecchymosis, necrotic papules, and hemorrhagic bullae.^1^^,^^6^ Other physical manifestations include solitary ulcers and suppurative nodules in a sporotrichoid (lymphangitic) pattern.^6^ Likewise, *S apiospermum* can present similarly on cytopathology and histopathology as *Aspergillus* spp, *Fusarium* spp, and *Petriella* spp.^6^ All these hyalohyphomycotic organisms produce hyaline hyphal structures that display septation at regular intervals and have dichotomous branching.^6^ *S apiospermum* may also be confused with yeasts, featuring terminal or intercalary chlamydospores.^6^ Thus, tissue cultures are vital to accurate speciation and identification. However, in the case of negative cultures because of empiric antifungal exposure, additional blood tests can be employed to supplement strong clinical suspicion.^7^ For example, negative serum galactomannan—a polysaccharide antigen almost found exclusively in the cell wall of *Aspergillus* spp—can be used to rule out the common pathogen.^7^ Additionally, positive serum 1,3-β-d-glucan—another cell wall component—can be used to alert clinicians of other fungal species, including *Aspergillus* spp, *Candida* spp, *Pneumocystis jirovecii*, and *Pseudomonas aeruginosa.*^7^ With a variety of tools at their disposal, dermatologists have the ability to improve the clinical outcomes of *S apiospermum* infection, especially in immunocompromised patients.

## Conflicts of interest

None disclosed.
